# SUMO-specific protease 1 protects neurons from apoptotic death during transient brain ischemia/reperfusion

**DOI:** 10.1038/cddis.2016.290

**Published:** 2016-11-24

**Authors:** Huijun Zhang, Yan Wang, Aoxue Zhu, Dehua Huang, Shining Deng, Jinke Cheng, Michael X Zhu, Yong Li

**Affiliations:** 1 Department of Biochemistry and Molecular Cell Biology, Shanghai Key Laboratory for Tumor Microenvironment and Inflammation, Institute of Medical Sciences, Shanghai Jiao Tong University School of Medicine, Shanghai 200025, China; 2 Department of Developmental and Behavioral Pediatrics, Shanghai Institute of Pediatric Translational Medicine, Shanghai Children's Medical Center, Shanghai Jiao Tong University School of Medicine, Shanghai 200025, China; 3 Department of Integrative Biology and Pharmacology, The University of Texas Health Science Center at Houston, Houston, TX 77030, USA

## Abstract

SUMO-specific protease 1 (SENP1) deconjugates SUMO from modified proteins. Although post-ischemic activation of SUMO conjugation was suggested to be neuroprotective against ischemia/reperfusion (I/R) injury, the function of SENP1 in this process remained unclear. Here we show that transient middle cerebral artery occlusion in mice followed by 6, 12 and 24 h reperfusion significantly enhanced SENP1 levels in the affected brain area, independent of transcription. Consistent with the increase in SENP1, the levels of SUMO1-conjugated proteins were decreased by I/R in cortical neurons of control littermate mice, but unchanged in that of animals with conditional ablation of SENP1 gene from adult principal neurons, the SENP1^flox/flox^:CamKII*α*-Cre (SENP1 cKO) mice. The SENP1 cKO mice exhibited a significant increase in infarct volume in the cerebral cortex and more severe motor impairment in response to I/R as compared with the control littermates. Cortical neurons from I/R-injured SENP1 cKO mice became more apoptotic than that from control littermates, as indicated by both TUNEL staining and caspase-3 activation. Overexpression of SENP1 in somatosensory cortices of adult wild-type (WT) mice suppressed I/R-induced neuronal apoptosis. We conclude that SENP1 plays a neuroprotective role in I/R injury by inhibiting apoptosis through decreasing SUMO1 conjugation. These findings reveal a novel mechanism of neuroprotection by protein desumoylation, which may help develop new therapies for mitigating brain injury associated with ischemic stroke.

Sumoylation is a post-translational modification that modifies the interaction of target proteins with protein partners and thereby alters their subcellular localization, activity and stability. The dynamic equilibrium between protein sumoylation and desumoylation is regulated by SUMO-specific proteases (SENPs). A total of six SENPs (SENP1–3 and SENP5–7) with different cellular distributions and SUMO paralogue specificities have been identified in mammals. By removing SUMO from sumoylated proteins, the SENPs not only reverse the modification but also yield a renewed source of free SUMO for conjugation to other proteins.^1^ Among the SENPs, SENP1 has a broad specificity for SUMO1 and SUMO2/3 and regulates both the maturation and deconjugation of these SUMO substrates.^2, 3^

A number of studies have shown increased SUMO conjugation of proteins in the brain of animal models of cerebral ischemia/stroke.^4, 5, 6^ This suggests that protein sumoylation may play a role in determining the fate of post-ischemic neurons. In a rodent model of transient middle cerebral artery occlusion (tMCAO), SUMO2/3-conjugated, but not SUMO1-conjugated proteins, were found to be increased in the hippocampus and cerebral cortex.^5, 6^ In animals subjected to permanent and transient focal cerebral ischemia, both SUMO1 and SUMO2/3 conjugations were shown to be activated not only in the infarcted striatum but also in the contralateral striatum.^4^ Moreover, SUMO1 conjugation was shown to be markedly activated in the state of hibernation torpor.^7^ It is unclear, however, under these conditions, whether and how the activity and expression of SENP1 are changed in conjunction with the alterations of SUMO-conjugated proteins.

Interestingly, in cultured hippocampal neurons subject to *in vitro* oxygen and glucose deprivation (OGD) treatment, not only the levels of SUMO-conjugated proteins but also the expression of SENP1 were increased, suggesting that the neuronal response to OGD may involve changes in both sumoylation and desumoylation.^3^ In addition, a blockade of SUMO2/3 translation in primary cortical neurons enhanced vulnerability to the OGD-induced damage, indicating that SUMO2/3 conjugation may be protective to neuronal injury.^8^ Moreover, sumoylation appeared to exert a role in ischemic preconditioning, an intrinsic process in which repeated short subtoxic episodes of ischemia protect against a subsequent major ischemic insult. Whereas overexpression of SUMO1 or SUMO2 in either cortical neurons or SHSY5Y cells increased survival following OGD, RNAi depletion of SUMO1 attenuated the effect of preconditioning.^9^ Furthermore, transgenic mice overexpressing Ubc9, which have elevated global sumoylation levels, also showed increased protection against focal cerebral ischemic damage.^10^ Therefore, existing evidence seems to point to an overall cytoprotective role of neuronal protein sumoylation during ischemic insult and preconditioning. However, little is known about the dynamics of sumoylation and desumoylation during this process. Particularly, despite the broad effect of SENP1 on both SUMO1- and SUMO2/3-conjugated proteins, how SENP1 contributes to the neuroprotective effect of sumoylation remained unexplored. Here, we selectively eliminated SENP1 from adult mouse neurons using conditional knockout (cKO) approach and examined its effects on brain damage induced by transient cerebral ischemia. Our results reveal an unexpected protective role of neuronal SENP1 in ischemia followed by reperfusion, which should inform new strategies for neuroprotection in ischemic stroke.

## Results

### SENP1 is increased after transient brain ischemia and reperfusion

**Figure 1:**
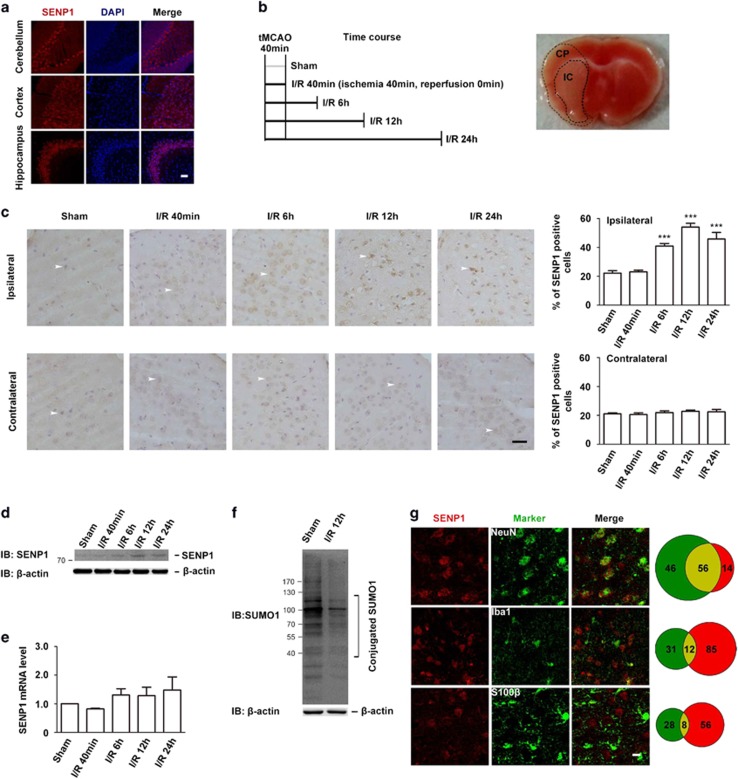
SENP1 expression is increased by ischemia/reperfusion. (**a**) Confocal images of immunohistochemical staining of SENP1 in coronal sections of adult mouse using an SENP1 antibody. Shown are representative images from at least three independent experiments. Scale bar, 20 *μ*m. (**b**) Schematic drawing of experimental design for brain ischemia (I) by tMCAO and reperfusion (R) (left) and a representative image of TTC-stained brain section from I/R-treated mouse (right). The cortical penumbra (CP) area is marked. This and equivalent areas in the contralateral hemisphere or sham-operated animals were used to determine the level of I/R injury in most subsequent experiments. Note: infarction core (IC). (**c**) Immunohistochemical detection of SENP1-positive cells (arrows) from the ipsilateral and contralateral hemispheres of sham-operated and tMCAO-operated WT mice with varying durations of reperfusion as indicated. Quantification of SENP1-positive cells (bar graphs at right, means±S.E.M. from three experiments with *n*=6, 4, 5, 5 and 4 mice for Sham, I/R 40 min, 6 h , 12 h  and 24 h, respectively) indicates increases in the ipsilateral hemisphere with >6 h of reperfusion, but no change in the contralateral hemisphere. ***P<0.001 compared with sham, by one-way analysis of variance with pair-wise comparison by Dunnett *post hoc* test. Scale bar, 50 *μ*m. (**d**) SENP1 protein levels were analyzed by western blotting in sham-operated and 40 min tMCAO-operated WT mice with varying durations of reperfusion as indicated. (**e**) SENP1 mRNA levels determined by quantitative real-time RT-PCR in cortices of WT mice subjected to treatments as indicated. The measured level of SENP1 mRNA was first normalized to that of GAPDH mRNA and then expressed relative to the SENP1 mRNA level in sham-operated control animals. Data are means±S.E.M. from four to six experiments with *n*=8, 7, 6, 5 and 5 mice for Sham, I/R 40 min, 6 h, 12 h and 24 h, respectively. *P*>0.05 compared with sham-operated, by one-way analysis of variance with pair-wise comparison by Dunnett *post hoc* test. (**f**) Western blot showing SUMO1-conjugated proteins in cerebral cortices of WT mice subjected to sham operation and I/R 12 h. I/R 12 h led to a decrease of SUMO1 conjugation. The blot is representative of three independent experiments. (**g**) Representative double immunofluorescence staining of SENP1 (red) and markers (green) for neurons (NeuN, top panels), microglia (Iba1, middle panels), or glia (S100*β*, bottom panels) in cortical areas of brain sections from 9- to 12-week-old WT mice. Statistics at the right side indicate 80.0% (56 of 70), 12.4% (12 of 97) and 12.5% (8 of 64) overlap of SENP1-positive staining with NeuN-, Iba1- and S100*β*-expressing cells, respectively. Conversely, 54.9% (56 of 102), 27.9% (12 of 43) and 22.2% (8 of 36) of NeuN-, Iba1- and S100*β*-expressing cells, respectively, are SENP1 positive, indicating that SENP1 is expressed primarily in neurons, with only a small fraction in glial cells in the cerebral cortex (*n*=4). Scale bar, 10 *μ*m

SUMO1/sentrin-specific peptidase 1 (SENP1) deconjugates SUMOs from modified proteins and is involved in processes such as nuclear transport, post-translational modification, transcriptional regulation, apoptosis and protein stability. To evaluate the role of SENP1 in neuronal damage following brain ischemia and reperfusion (I/R), we first examined the expression of SENP1 in major brain areas of C57BL/6 mice, including cerebellum, cortex and hippocampus. Immunohistochemical analysis revealed that SENP1 is expressed in these brain regions (Figure 1a). With 40 min tMCAO followed by a reperfusion for 0, 6, 12 or 24 h, as outlined in Figure 1b (left), a large area of brain infarction developed in the ipsilateral hemisphere, which was detected by straining brain sections with 2,3,5-triphenyltetrazolium chloride (TTC; Figure 1b, right). The TTC staining reveals the cortical penumbra (CP) as labeled by the contour lines in Figure 1b (right). CP was used here and in most of the subsequent experiments to determine the level of I/R injury. Consistent with the previous study,^11^ immunohistochemical staining of randomly selected CP areas showed that the percentages of SENP1-positve cells in sections from wild-type (WT) mice were significantly increased at 6, 12 and 24 h reperfusion in the ipsilateral hemisphere, but not in the equivalent areas of the contralateral hemisphere (Figure 1c). Western blot analysis also revealed increased SENP1 protein levels in the ipsilateral hemisphere over these time periods (Figure 1d). However, using similar samples as that used for western to perform quantitative RT-PCR, we only detected moderate changes in SENP1 mRNA levels, which did not reach statistical significance (Figure 1e), suggesting that transcriptional mechanism did not play a major part in the upregulation of SENP1.

While some previous studies reported no major change of SUMO1-conjugated proteins in response to transient forebrain ischemia, despite the increased levels of SUMO2/3 conjugation,^6, 12^ others observed changes in the pattern of SUMO1-conjugated proteins depending on the duration of OGD treatment.^9, 13, 14^ To address whether SUMO1-conjugated proteins were changed in our model, we compared SUMO1-conjugated proteins in WT mice that had been either sham operated or subjected to 40 min tMCAO followed by 12 h reperfusion and found the levels of SUMO1-conjugated proteins to be significantly decreased in brain samples prepared from animals subjected to I/R injury (Figure 1f). Supporting a neuronal function of SENP1, SENP1 showed abundant expression primarily in neurons, with only a small fraction in glial cells (Figure 1g). Taken together, these results indicate that SENP1 expression levels in principal neurons are increased in response to I/R injury and this leads to a decrease in protein SUMO1 conjugation.

### Neuron-specific SENP1 cKO revealed selective regulation of SUMO1 but not SUMO2/3 conjugation by SENP1 during I/R injury

**Figure 2:**
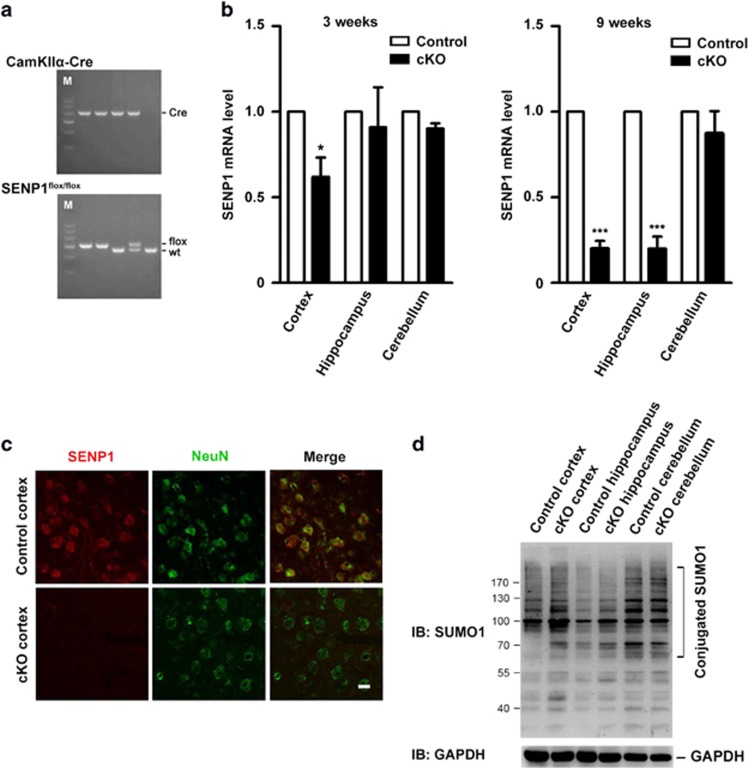
Generation of neuron-specific SENP1 cKO mice. (**a**) Representative genotyping results for identification of SENP1 cKO mice by PCR. (**b**) The relative SENP1 mRNA levels in the cortices, hippocampi and cerebella of SENP1 cKO mice normalized to that in corresponding areas of control littermates at 3 (left) and 9 (right) weeks, measured by quantitative real-time RT-PCR. SENP1 mRNA in the cKO mice was reduced at 3 and 9 weeks in the cortex, at 9 weeks in the hippocampus, but not changed in the cerebellum, where Cre is not expressed. Data shown are means±S.E.M. from three experiments with *n*=4 mice in each category (control littermates and cKO mice, 3 and 9 weeks). *P<0.05, ***P<0.001 compared with controls, by Student's *t*-test. (**c**) Double immunofluorescence staining of brain sections from 9-week-old SENP1 cKO and littermate control mice using antibodies against SENP1 (red) and NeuN (green), confirming the loss of SENP1 expression in cortical neurons. Images are representative of at least four independent experiments. (**d**) Western blot analysis of lysates derived from cerebral cortices, hippocampi and cerebella of 9-week-old SENP1 cKO and littermate control mice, using an anti-SUMO1 antibody. As expected, the levels of SUMO1-conjugated proteins were increased in cerebral cortices and hippocampi, but not in cerebella, of SENP1 cKO mice. GAPDH was used as a loading control. The blot is representative of three independent experiments

Global deletion of SENP1 causes anemia and embryonic lethality between E13.5 to postnatal day 1.^15^ Thus, we took advantage of the recently available SENP1^flox/flox^ mouse line^16^ for cKO to create SENP1^flox/flox^:CamKII*α*-Cre mice,^17^ in which SENP1 was selectively removed from principal neurons of postnatal forebrain (Figure 2a). This allowed us to specifically address SENP1 function in the postnatal forebrain without disrupting its contribution to early central nervous system development and/or causing embryonic lethality. In the cKO mice, SENP1 mRNA and protein levels were strongly reduced in the forebrain as determined by quantitative real-time RT-PCR and immunofluorescence staining (Figures 2b and c). The SENP1 mRNA began to decrease in the cortex at 3 weeks after birth and reached >80% reduction in hippocampus and cortex by 9 weeks in the cKO mice (Figure 2b). No change was detected in the cerebellum where CamKII*α*-Cre is not expressed (Figure 2b). Additionally, western blot analysis revealed elevations of SUMO1-conjugated proteins in extracts isolated from the cortices and hippocampi, but not from the cerebella, of 9-week-old SENP1^flox/flox^:CamKII*α*-Cre (SENP1 cKO) mice (Figure 2d).

**Figure 3:**
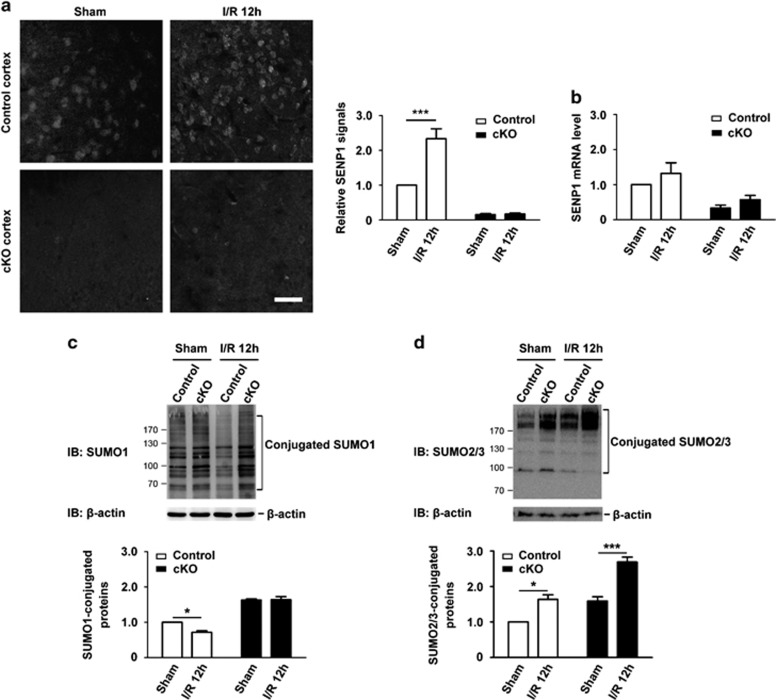
SENP1 deficiency in neurons abolishes decreased SUMO1 conjugation but not increased SUMO2/3 conjugation in response to ischemia/reperfusion. (**a**) Immunofluorescence staining of SENP1 in coronal sections of cerebral cortices from SENP1 cKO and littermate control mice subjected sham operation and 40 min ischemia followed by 12 h reperfusion. While a robust increase was seen in I/R 12 h-treated control littermates, no alteration was found in SENP1 cKO mice. Images are representative of at least three independent experiments, which are summarized in the bar graph at right. Scale bar, 50 *μ*m. (**b**) Comparison of SENP1 mRNA levels determined by quantitative real-time RT-PCR in the cortices of littermate control and SENP1 cKO mice subjected to treatments as indicated. The measured level of SENP1 mRNA was first normalized to that of GAPDH mRNA and then expressed relative to the SENP1 mRNA level in sham-operated animals. Data are means±S.E.M. from four to six experiments with *n*=6–7 for littermate control and *n*=4–5 for cKO mice. *P*>0.05 compared with the corresponding sham-operated control, by Student's *t*-test. (**c** and **d**) Representative western blots showing SUMO1 (**c**) and SUMO2/3 (**d**) conjugated proteins in cerebral cortices of littermate control and SENP1 cKO mice subjected to sham operation and I/R 12 h. Quantification data (means±S.E.M. from three experiments with *n*=5 mice each for littermate control and cKO in the Sham group and *n*=4 littermate control and 3 cKO mice in the I/R 12 h group) for overall cortical levels of SUMO1- and SUMO2/3-conjugated proteins, normalized to that in sham-operated control littermates are shown below. The levels of SUMO1-conjugated proteins were increased in SENP1 cKO mice no matter if the animals were subjected to I/R or not; I/R 12 h led to a decrease of SUMO1 conjugation in control littermates, but not in the cKO mice; the levels of SUMO2/3-conjugated proteins were increased in both SENP1 cKO mice and control littermates following I/R. *P<0.05, ***P<0.001

In cortices of SENP1 cKO mice, which still expressed SENP1 in non-neuronal cells, tMCAO and 12 h reperfusion did not induce a change in SENP1 expression at either protein (Figure 3a) or mRNA (Figure 3b) levels. As expected from the loss of a desumoylating enzyme, the overall levels of SUMO1-conjugated proteins were increased in the cKO brains, as compared with the littermate controls, no matter if the animals were subjected to I/R or not (Figure 3c). With the injury, the levels of SUMO1-conjugated proteins were significantly decreased only in the littermate controls but not the cKO brains when compared with the corresponding sham-operated samples (Figure 3c). However, the levels of SUMO2/3-conjugated proteins were significantly increased in brains from both the littermate controls and SENP1 cKO mice in response to 40 min tMCAO followed by 12 h reperfusion (Figure 3d). This is consistent with the previous reports showing increased protein SUMO2/3 conjugation in hippocampal and cortical areas in both WT rat and mouse models of tMCAO.^5, 6^ The similar increase observed in the SENP1 cKO mouse brains as compared with the littermate controls suggests that SENP1 is not critically involved in regulating SUMO2/3 conjugation in neurons of the I/R-injured brain.

### SENP1 deficiency exacerbates the tMCAO-induced brain damage and neurological deficits

**Figure 4:**
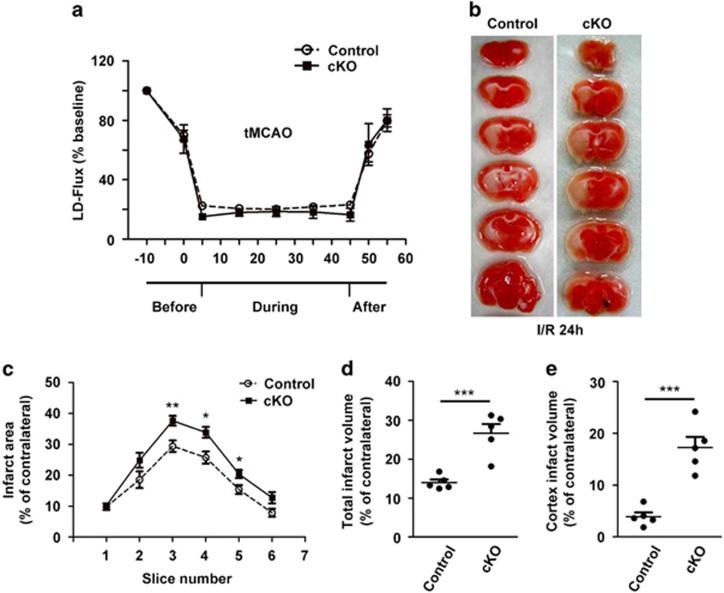
SENP1 deficiency exacerbates ischemic brain damage. (**a**) Regional cerebral blood flow (rCBF) in the core of the middle cerebral artery territory of SENP1 cKO and littermate control mice before, during and after ischemia measured by laser Doppler flowmetry. Flow values from seven experiments are averages of 10 min intervals and are expressed as means±S.E.M. No difference was noticed between littermate control and SENP1 cKO mice. (**b**) Photomicrographs of TTC-stained brain sections (1 mm) showing brain infarction at 24 h after the onset of ischemia in littermate control and SENP1 cKO mice. Representative of five experiments for each. (**c–e**) Quantification of infarct areas (volumes) in ipsilateral hemisphere normalized to the total areas (volumes) of the corresponding contralateral hemisphere in sequential coronal brain slices (**c**), the entire brain (**d**) and the cortical regions (**e**). For (**d**) and (**e**), results for individual animals are shown as dots and means±S.E.M. are indicated as horizontal lines *P<0.05, **P<0.01, ***P<0.001 by Student's *t*-test

To determine the functional significance of neuronal SENP1 in ischemic brain injury, we compared the infarct areas of SENP1 cKO and the littermate control mice that underwent tMCAO for 40 min followed by 24 h reperfusion. To ensure a similar degree of ischemic insult to the brain, cerebral blood flow was measured for each mouse by laser Doppler flowmetry (LDF) during tMCAO (Figure 4a). At 24 h after reperfusion, the mouse was killed and coronal sections of the brain were prepared and stained with TTC to visualize the damaged brain regions for determination of infarct volume. Quantification of the TTC-stained sections showed that infarct areas of SENP1 cKO mice were significantly larger than that of littermate controls (Figures 4b–d). Particularly, the infarct areas in the cortices of SENP1 cKO mice were about four times larger than that of the control littermates (Figure 4e). These results suggest that neuronal SENP1 proteins are protective against the damage caused by transient ischemic insult and/or subsequent reperfusion.

**Figure 5:**
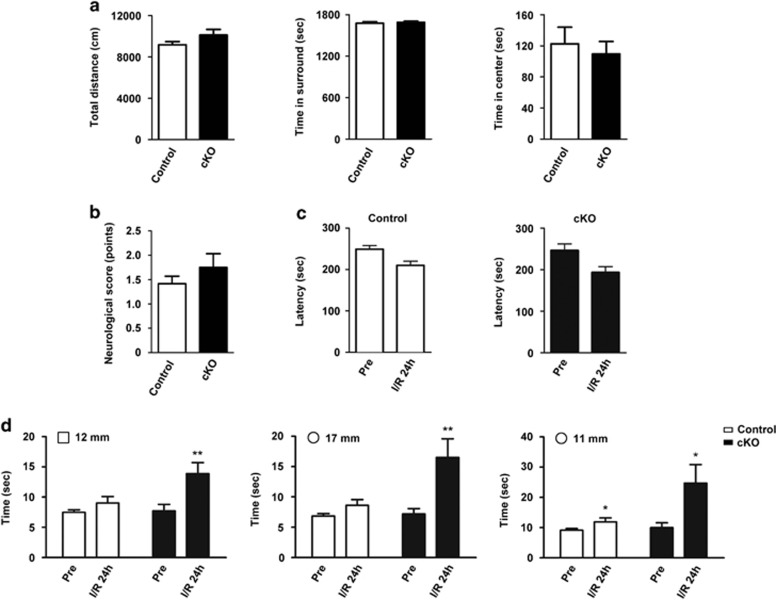
SENP1 deficiency exacerbates motor control deficits in response to brain ischemia/reperfusion. (**a**) Locomotor activity and exploratory behavior of uninjured 9- to 12-week old SENP1 cKO mice and their control littermates assessed by open-field test. Quantifications of total distance moved (left), time spent in surrounding areas (middle) and in the center (right) were made for 30 min during each session. Data are means±S.E.M. for 20 littermate control and 12 SENP1 cKO mice. *P*>0.05 by Student's *t*-test for all three parameters. (**b**) Neurological deficits of SENP1 cKO and littermate control mice subjected to I/R 24 h. The neurological deficit scores were assessed based on a scale of 0 to 4 (see Materials and Methods) and data (means±S.E.M.) were pooled from six experiments with a total of six littermate control and six cKO mice. *P*>0.05 by Student's *t*-test. (**c**) Rotarod test. SENP1 cKO and littermate control mice were tested for three consecutive trials on the rotarod before operation (pre) and after I/R 24 h. Data represent means±S.E.M. of time spent on the rotarod until falling of 19 littermate control and 11 SENP1 cKO mice for pre, and 13 littermate control and 10 SENP1 cKO mice for I/R 24 h pooled from two independent experiments. (**d**) Beam walking test. SENP1 cKO and littermate control mice were tested for balance beam walking on 12-mm square (left) and 17-mm (middle) and 11-mm (right) round beams before operation (pre) and after I/R 24 h. The time it took for the mouse to cross the beam (80 cm distance) was recorded. Data are means±S.E.M. of 19 littermate control and 11 cKO mice for pre-operation and 13 littermate control and 10 cKO mice for I/R 24 h pooled from two independent experiments. *P<0.05, **P<0.01, by Student's *t*-test

Next, we examined whether neuron-specific deletion of SENP1 in adult mice altered neurological functions in uninjured and injured animals. To evaluate the possible presence of any inherent neurological deficit, the uninjured SENP1 cKO mice and their control littermates were assessed using open-field test, which revealed no significant difference between genotypes (Figure 5a). Following the ischemic injury and subsequent reperfusion, the neurological deficits were assessed by a series of behavioral tests and their neurological performance scored based on a scale of 0 to 4 (see Materials and Methods). Both SENP1 cKO and the littermate control mice showed neurological deficits, but no significant difference was found in the neurological deficit scores between them despite a tendency for the cKO mice to perform worse than the control littermates (Figure 5b). The latency to fall on the rotarod test was moderately decreased for either genotype, but no difference was found between the cKO and littermate control mice (Figure 5c). However, in a modified beam balance test, which further assesses the motor skills before and after the ischemic insult (40 min tMCAO) followed by 24 h reperfusion, we found that while the control littermates only displayed a moderate increase in the time required to cross the 11 mm round beam, but not that needed to travel through the 12 mm square and 17 mm round beams, the SENP1 cKO mice took a significantly longer time to cross each of the three beam types after the injury than before the injury (Figure 5d). This indicates more severe declines in the motor function in the injured cKO mice than their control littermates. Together, these observations suggest that the neuron-specific deletion of SENP1 in adult mice can exacerbate ischemic brain damage, worsening the neurological deficits.

### SENP1 deficiency exacerbates cortical neuron apoptosis in response to I/R injury

**Figure 6:**
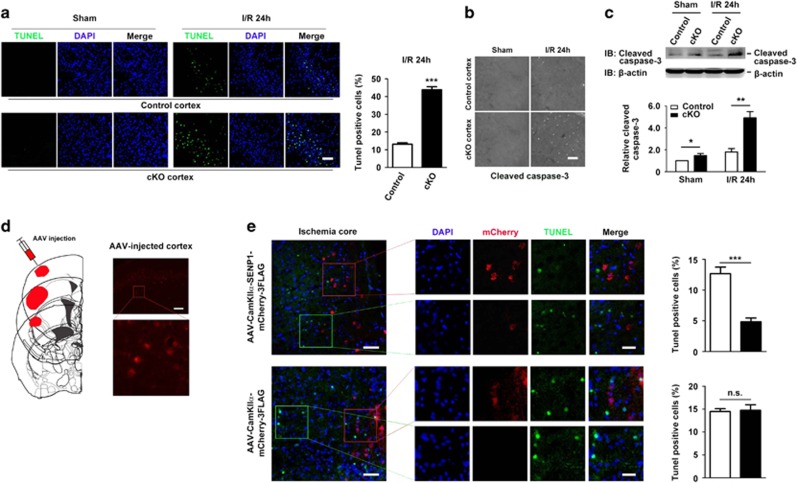
SENP1 deficiency exacerbates cortical neuron apoptosis in response to brain ischemia/reperfusion. (**a**) Representative images of TUNEL and DAPI co-staining of cortical areas of brain sections of littermate control and SENP1 cKO mice subjected to sham operation (left) or 40 min ischemia plus reperfusion for 24 h (I/R 24 h, right). Scale bar: 50 *μ*m. Quantification of TUNEL-positive cells in littermate control and cKO mice is shown to the right, as means±S.E.M. for seven mice each from three experiments. ***P<0.001, by Student's *t*-test. (**b** and **c**) Immunohistochemistry (**b**) and western blotting (**c**) of activated caspase-3 in cortical areas of littermate control and SENP1 cKO mice subjected to sham operation or I/R 24 h. Note the obvious increase in cleaved caspase-3-positive cells in the cKO as compared with littermate control samples after I/R 24 h in (**b**). Images in (**b**) are representative of at least three independent experiments with *n*=5 littermate control and 6 cKO mice in the Sham group and *n*=5 littermate control and 5 cKO mice in the I/R 24 h group. Scale bar, 100 *μ*m. Bar graph in (**c**) shows quantification of activated caspase-3 protein, normalized to the level in sham-operated control littermates, based on western blots exemplified above. *β*-Actin was used as a loading control. Data are means±S.E.M. from four experiments with *n*=4 littermate control and 4 cKO mice in the Sham group and *n*=6 littermate control and 5 cKO mice in the I/R 24 h group. *P<0.05, **P<0.01 by Student's *t*-test. (**d**) Schematic representation of the AAV viral infection into the primary somatosensory cortex, barrel field and secondary somatosensory cortex of WT mice (left), with a representative brain image showing the expression of mCherry in cortex (right). The boxed area is enhanced by high magnification to show mCherry-positive neurons. Scale bar: 100 *μ*m. (**e**) Representative images of cortical areas of brain sections of virus-infected WT mice subjected to 40 min ischemia plus reperfusion for 24 h, co-labeled for TUNEL, mCherry and DAPI. Mice were infected with AAV-CamKII*α*-SENP1-mCherry-3FLAG (upper) or the vector control, AAV-CamKII*α*-mCherry-3FLAG (lower). Middle panels show high-magnification views of the red and green boxes, representing areas with abundant and low expression, respectively, of SENP1-mCherry or control mCherry. Quantification (means±S.E.M. for three mice each from three experiments) of TUNEL-positive cells in the abundant (black bars) and low (white bars) expression ischemic zones are shown at right. ***P<0.001, by Student's *t*-test. Scale bars: 50 and 25 *μ*m for left and middle panels, respectively

To investigate the cellular mechanism of I/R-induced brain damage and neurological deficits associated with SENP1 deficiency, we performed TUNEL assay to assess the apoptotic like neurons in brain slices from SENP1 cKO and littermate control mice subjected to 40 min tMCAO and 24 h reperfusion. Although nearly no TUNEL-positive cell was found in cortical areas of sham-operated mice of either genotype, significantly more TUNEL-positive cells were found in cortical areas on the ischemic side of the SENP1 cKO mice than the control littermates (Figure 6a). The majority of TUNEL staining occurred in the nuclei, as shown by the co-labeling with DAPI, a nuclear marker (Figure 6a). Because caspase-3 activation has been implicated in neuronal damage associated with focal cerebral ischemia,^18, 19^ we also performed immunohistochemistry and western blotting for the activated, cleaved, form of caspase-3. As compared with samples from the littermate control mice, the number of caspase-3-positive cells was increased in ischemic cortical areas of the SENP1 cKO mice (Figure 6b); the amount of cleaved caspase-3 in the ischemic cortices was also robustly elevated in the SENP1 cKO mice as revealed by western blotting (Figure 6c). Collectively, these results suggest a protective function of neuronal SENP1 against apoptosis of cortical neurons in response to I/R.

To further validate the antiapoptotic role of neuronal SENP1 in response to I/R, we examined if overexpression of SENP1 in WT cortical neurons could be neuroprotective. To this end, we injected adeno-associated viruses (AAV) coding for SENP1-mCherry-3FLAG or the vector control mCherry-3FLAG into the primary somatosensory and secondary somatosensory cortices of adult WT mice (Figure 6d, left). As shown by fluorescence microscopic imaging (Figure 6d, right), 4 weeks after stereotactic intracerebral injection, the virus-mediated expression, based on the red mCherry fluorescence, was readily detectable in the primary and secondary somatosensory cortex. As expected, the virus-mediated SENP1 gene transfer compensated for neuronal apoptosis in response to I/R. The occurrence of TUNEL-positive cells in areas with high SENP1 (mCherry) expression was significantly less than that with low SENP1 (mCherry) expression (Figure 6e, upper panel). By contrast, the vector control virus did not produce such an effect (Figure 6e, lower panel), ruling out the possibility that protection might be caused by an SENP1-independent unspecific effect of virus transduction. These data further support that SENP1 plays an important function against apoptosis of cortical neurons in response to I/R.

## Discussion

Growing evidence has implicated the importance of sumoylation in regulation of cell signaling, protein stability and apoptosis.^2, 20^ However, whether and how sumoylation contributes to neuronal damage in the brain in response to stroke-like I/R injury remain poorly understood. Here, we present a new mouse model with neuron-specific deletion of the gene for a desumoylating enzyme, SENP1, using Cre/LoxP-mediated method. We found that I/R injury caused more severe brain damage to the SENP1 cKO mice than control littermates, including larger infarct volume, stronger motor deficits and a greater number of apoptotic neurons. These suggest that hyper-sumoylation resulting from disruption of SENP1 is detrimental to the neuron under ischemic stress and the likely primary cause of neuronal death is the enhanced apoptosis, which can worsen brain injury and neurological deficits. In addition, SENP1 overexpression protected against cell death in I/R injury. Therefore, SENP1-mediated protein desumoylation has an antiapoptotic function, pivotal for neuronal cell survival during the process of brain I/R.

Previous studies have shown that SUMO1 or SUMO2/3-conjugated proteins are globally elevated after I/R and the altered protein sumoylation is believed to play a major effect on the fate of post-ischemic neurons.^4, 5, 6^ However, sumoylation is a common post-translational modification that occurs to many proteins. It is likely that both pro-death and pro-survival pathways are activated by sumoylation depending on the type of the protein and the time when the protein becomes sumoylated and/or desumoylated. Therefore, a global change of the overall sumoylation level in the tissue, or even in a population of cells, gives no indication whether the modification would be beneficial or detrimental.

In fact, it remains unresolved whether the overall levels of SUMO1-conjugated proteins are altered in the ischemic brain or neurons subjected to OGD treatment. While some studies reported no major change in SUMO1-conjugated proteins in the forebrain after transient ischemia, despite the increased levels of SUMO2/3 conjugation,^6, 12^ others noticed changes in the pattern of SUMO1 conjugation of proteins depending on the duration of OGD treatment.^9, 13, 14^ Therefore, SUMO1-conjugated proteins likely undergo dynamic changes during the process of I/R and the resulting patterns may differ because of the models used and the treatment times applied. In our hands, the 40 min tMCAO followed by 12 h reperfusion actually led to reduced overall SUMO1 conjugation of proteins in cortical neurons of WT mice (Figures 1f and 3c), which, although unexpected based on previous studies,^5^ is consistent with the current results showing increased SENP1 expression in response to the injury (Figure 1). In addition, the lack of SENP1 prevented the injury-induced decrease in SUMO1 conjugation, but not the injury-induced increase in SUMO2/3 conjugation, indicating that SENP1 upregulation and the resultant selective desumoylation of SUMO1-conjugated proteins constitute an important step of neuroprotection in response to ischemic insult. Although we cannot exclude that with different severities of ischemia, varying durations of ischemia and reperfusion, or different methods to induce the ischemic injury, the pattern of SUMO1 conjugation changes may differ, it is clear that the conjugations by SUMO1 and SUMO2/3 have distinct functions in ischemic brain injury, with SUMO1 conjugation being more common under normal conditions whereas SUMO2/3 conjugation upregulated during I/R insult.

It has been suggested that apoptosis plays a critical role in neuronal death in the ischemic penumbra after focal cerebral ischemia.^21, 22^ Whether neurons die by apoptosis or necrosis after injury depends on the severity of injury.^23^ A number of studies have demonstrated the presence of apoptotic neurons using the TUNEL assay and caspase-3 staining as indications of hypoxic or ischemic neuronal death in a variety of tissues, such as peripheral nerves, ganglia and the retina.^24, 25, 26, 27, 28^ It is well established that apoptosis can be triggered by a number of factors, such as oxidative stress and mitochondrial dysfunction. Caspase-3 activation is considered as the final step and common pathway of multiple apoptotic cascades.^29^ Cerebral ischemia may enhance some of these and thereby lead to increased apoptosis.^30^ Indeed, we detected increases in the numbers of TUNEL-positive cells and cleaved caspase-3 in the forebrain of littermate control mice subjected to 40 min ischemia followed by 24 h reperfusion, which in turn were reduced by virus-mediated SENP1 (mCherry) expression in cortical neurons (Figure 6). Importantly, these increases were more robust in the forebrain of SENP1 cKO mice, suggesting that the desumoylating action of SENP1 in cortical neurons is protective against apoptotic cell death.

Several lines of evidence suggest that the dynamic regulation of sumoylation serves as a cytoprotective pathway in neurons. Indeed, the desumoylating enzyme, SENP3, is rapidly degraded during OGD, allowing prolonged SUMO2/3 modification of Dynamin-related protein 1 and its sequestration from mitochondria.^20, 31^ Following reoxygenation, SENP3 expression recovers, which reverses the protective effect of SUMO2/3 modification and promotes cell death.^20^ However, this *in vitro* result on SENP3 expression and SUMO2/3 conjugation is inconsistent with that obtained from *in vivo* I/R studies, which showed persistent increases in the levels of SUMO2/3-conjugated proteins in brains subjected to tMCAO followed by reperfusion for 0.5 to 6 h.^5, 6^ While we confirmed that protein SUMO2/3 conjugation in mouse brain was indeed increased due to *in vivo* I/R injury after 12 h reperfusion, which was unaffected by SENP1 deletion in neurons, we found that protein SUMO1 conjugation was decreased under the same conditions and this required neuronal expression of SENP1 (Figures 1f, 3c and 3d). It remains to be illustrated the detailed time course of SUMO1 conjugation changes during ischemia and reperfusion and how SENP1 affects it during different stages of I/R. Previously, conjugation by ectopically expressed SUMO1 in transgenic mice was shown to be dramatically decreased during 10 min tMCAO and then increased at 1 h and 3h of reperfusion, but decreased again at 6 h of reperfusion.^32^ To what extent the 40 min tMCAO differs from the 10 min ischemia and if endogenous SUMO1 behaves the same as the ectopically expressed tagged SUMO, as well as whether all these changes depend on SENP1 are subjects of future studies. Nonetheless, accumulating evidence has shown that the stability and activity of the enzymatic components of SUMO system must be tightly and dynamically regulated during the processes of I/R.

Therefore, the simple model of a beneficial role suggested for enhanced sumoylation in post-ischemic injury needs to be refined, as desumoylation by SENP1 can be protective as well. Instead, a more complex picture has emerged, indicating that timely controls of finely balanced sumoylation of specific substrates rather than bulk conjugation/deconjugation of SUMO are critical. In our case, the data show clearly that in response to injury, SENP1 is upregulated in cortical neurons and this serves to reduce conjugation by SUMO1, but not SUMO2/3, and in turn suppresses caspase activation and cell death.

In conclusion, using the newly developed SENP1 cKO mice, we demonstrate a protective effect of SENP1 on post-ischemic neuronal cell damage. This represents the first report on the use of neuron-specific deletion of SENP1 in a mouse stroke model to determine the consequences of hyper-SUMO conjugation on neuronal survival in response to cerebral ischemia. Our results strongly suggest the importance of dynamic balance between sumoylation and desumoylation in neuronal fate determination during ischemic insults, establishing a critical link between SUMO conjugation and viability and function of post-ischemic neurons. Verifying the protective role of SENP1 in these neurons will aid the design of new strategies for preventive and therapeutic interventions in clinically relevant pathological states associated with ischemic stroke.

## Materials and methods

### Generation of neuron-specific SENP1^flox/flox^:CamKII*α*-Cre mice

All animal procedures were carried out in accordance with the guidelines for the Care and Use of Laboratory Animals of Shanghai Jiao Tong University School of Medicine and approved by the Institutional Animal Care and Use Committee. The mouse strains used in this study were generated and maintained on the C57BL/6 background. Adult male animals were used for experiments. SENP1^flox/flox^ mice, as described previously,^16^ were crossed with CamKII*α*-Cre mice^17^ to create neuron-specific cKO SENP1^flox/flox^:CamKII*α*-Cre mice. Littermate SENP1^flox/flox^ and CamKII*α*-Cre showed similar stroke responses and were therefore included as littermate controls unless indicated otherwise. PCR primers used for genotyping are: loxP Forward: 5′-AGAGTGAGACCCTGTCTCAACCCAAGC-3′ and loxP Reverse: 5′-CACACAACTAAGTTAACTGCTGGAAACCAGAGC-3′, with the expected product sizes of 300 and 260 bps for SENP1^flox/flox^ and WT mice, respectively. The presence of CamKII*α*-Cre was verified by PCR using primers: Cre Forward: 5′-CGCTGGGCCTTGGGACTTCAC-3′ and Cre Reverse: 5′-CAGCATTGCTGTCACTTGGTC-3′, with the expected product size of 403 bps.

### Transient middle cerebral artery occlusion

Male C57BL/6, SENP1^flox/flox^ and SENP1 cKO (9–12 weeks, 22–25 g) were housed under a 12 h light/dark cycle with free access to food and water throughout. Mice were anesthetized with pentobarbital sodium. Body temperature was maintained at 37 °C by a heating pad during the entire procedure. tMCAO was induced using intraluminal suture as previously described^33^ with minor modifications. Briefly, the left common carotid artery and the external carotid artery were exposed by a ventral midline neck incision and clipped. The external carotid artery was ligated with 5-0 silk suture. A 2-cm length of silicon-rubber-coated monofilament (7-0) was advanced from the common carotid through the internal carotid up to the level of the anterior cerebral artery. The suture was inserted 9–11 mm from the bifurcation of common carotid to occlude the middle cerebral artery. After 40 min of tMCAO, the suture was gently retracted to allow reperfusion for various durations (6, 12 and 24 h). LDF (Moore Instruments Limited, Devon, UK) was used to monitor regional cerebral blood flow from 15 min before to 10 min after tMCAO. Ischemia and reperfusion were defined as a minimum of 75% decrease in cerebral blood flow at the onset of ischemia and a return to 50% of baseline blood flow measurement, respectively. Mice not achieving these standards were excluded from the study. Among the 320 male animals that underwent the surgery for tMCAO, 80 of the ischemic animals were excluded because of insufficient decrease in cerebral blood flow following tMCAO (46), death during anesthetic induction, before completion of the surgery, during early reperfusion, or several hours before the 24 h post-MCAO study end point (28), or lack of obvious infarct lesion at 24 h after tMCAO (6). For infarct volume analysis each group consisted of five animals. Owing to animal deaths before the 24 h study end point, 10–13 animals per group were available for behavioral testing.

### Immunohistochemistry

Eight-micrometer-thick consecutive coronal sections from paraffin-embedded tissues deparaffinized and rehydrated before heat-induced antigen retrieval was conducted at pH 6.0 in 0.01 mol/l sodium citrate buffer in a steamer for 30 min to intensify staining. Immunostaining was performed as described^34^ with minor modifications. Sections were rinsed in 0.1 M phosphate-buffered saline (PBS, pH 7.4) with 0.2% Triton X-100, incubated with 0.3% hydrogen peroxide for 10 min to quench endogenous peroxidase activity and blocked with normal goat serum for 60 min at 37 °C. Sections were then incubated with the primary antibody (rabbit anti-SENP1 1:100; Abcam, Cambridge, MA, USA or IMGENEX, Littleton, CO, USA IMG-521) overnight at 4 °C. A secondary goat anti-rabbit biotinylated (1:400; Dako, Santa Clara, CA, USA) antibody was applied for 30 min at 37 °C, and sections were incubated with streptavidin-horseradish peroxidase conjugate (1:200; Invitrogen, Carlsbad, CA, USA) for 30 min. Quantitative analysis of SENP1-positive cells in the infarct cortical area and the corresponding area on the contralateral hemisphere were performed in a masked fashion in five random non-overlapping fields. The percentage of SENP1-positive cells was calculated using the ratio of SENP1-positive cells (brown color) over the total number of cell nuclei (blue color) counted × 100. Counts were made by an investigator blinded to the conditions of the experiments.

### Immunofluorescence staining

Immunofluorescence staining was performed as described previously.^35, 36^ Mice were perfused intracardially with 4% paraformaldehyde and postfixed overnight in 4% paraformaldehyde at 4 °C, cryoprotected in 30% sucrose and embedded in OCT. Tissues were cryosectioned at 15 *μ*m and mounted on slides before incubation with primary antibodies (SENP1, 1:100, IMG, Littleton, CO, USA; Iba1, 1:100, Abcam; S100*β* 1:100, Sigma, St. Louis, MO, USA; cleaved caspase-3, 1:100, CST, Danvers, MA, USA) overnight at 4 °C and then with Alexa Fluor546-conjugated goat anti-rabbit or Alexa Fluor488-conjugated goat anti-mouse antibodies (Invitrogen; 1:200) for 1 h at room temperature. Before incubating with primary antibodies, sections were rinsed in 0.1 M PBS (pH 7.4) with 0.2% Triton X-100 and blocked in goat serum for 1 h at room temperature. Sections were analyzed by epifluorescence microscopy (OLYMPUS IX71, Shinjuku-ku, Tokyo, Japan) or confocal fluorescence microscopy (Zeiss, Oberkochen, Germany, LSM 510).

### Infarct volume measurement

The volume of ischemic damage was calculated as described previously.^37^ At 24 h after tMCAO, animals were anesthetized, brains removed and sliced into six 1-mm-thick sections. The sections were incubated for 30 min at 37 °C in 2% TTC in saline and scanned on a cannon scanner. In each slice, the total area in the contralateral side and the noninfarcted area in the lesioned side were measured by an investigator blinded to the genotype using ImageJ. The areas on each side were summed over the number of sections evaluated, and the respective volumes were calculated by multiplying each sum by 1 mm (thickness of each section). The percentage of infarction volume was calculated as follows: ((volume of contralateral side−noninfarcted volume of the lesioned side)/volume of contralateral side) × 100% the percentage of cortex infarction volume: (volume of infarcted cortex of lesioned side/volume of total cortex of contralateral side) × 100%.

### Neurological deficiency assessment

A neurological score was assessed according to the scoring system described previously with minor modifications.^38^ An investigator who was blinded to the genotypes of the mice performed the analyses based on the following four-tiered grading system: 0=no deficit; 1=flexion of contralateral torso and forelimb upon lifting of the whole animal by the tail; 2=circling to the contralateral side, when held by tail with feet on floor; 3=spontaneous circling to contralateral side; 4=no spontaneous motor activity.

### Behavioral studies

Locomotor activity was measured using open-field test as described previously.^39^ Mice were implanted with fiber optics, then placed in the open-field apparatus and allowed to explore for 30 min. Total distance traveled and time spent in the center square was measured by a video-tracking system (EthoVision XT 8.0; Noldus Technology, Wageningen, The Netherlands).

### Motor outcome tests

To assess behavioral deficits before and after I/R, rotarod test was performed as described previously with minor modifications.^40^ The rotarod was set to start at 4 m/s and to accelerate to 44 m/s over 300 s.

### Beam walking test

The apparatus used in this experiment was modified from that described previously by Allbutt and Henderson (2007).^41^ Before tMCAO, mice were trained to transverse a 100 cm long and 12 mm wide wooden square beam suspended 50 cm above the floor in two consecutive training sessions with 3 h intervals each day for 3 days. Then, littermate control and SENP1 cKO mice, pre-operation and after 40 min ischemia with 24 h reperfusion, were subjected to trials on round (17 and 11 mm diameters) and square (12 mm diameters) beams. The time to reach a distance of 80 cm was measured. The same animals were used in both rotarod and beam walking tasks.

### Reverse transcription-polymerase chain reaction

Total RNA was extracted from uninjured or ischemic CP tissues using TRIzol reagents (Tiangen, Beijing, China) and reverse transcribed to obtain single-strand cDNA using a Reverse Transcription System (Takara, Shiga, Japan) as previously described.^42^ Reactions were performed in a 20 *μ*l final volume with Power SYBR Green PCR Master Mix (CWBIO, Beijing, China) and 0.2–0.5 *μ*M primers using the Applied Biosystems 7500 fast Real-Time PCR System (Foster City, CA, USA) according to the manufacturer's instructions. Results are the average of at least three independent biological replicates (one mouse from each of three different litters), each performed in triplicates. Primers used are: WT mSENP1: Forward: 5′-CTGGGGAGGTGACCTTAGTGA-3′ and Reverse: 5′-GTGATAATCTGGACGATAGGCTG-3′ cKO mSENP1: Forward: 5′-GTGAAACGCTGGACAAAGAAG-3′ and Reverse: 5′-GTCTACAACAGCTAGACACCAG-3′ mGAPDH: Forward: 5′-CATGGCCTTCCGTGTTCC-3′ and Reverse: 5′-GCCTGCTTCACCACCTTCTT-3′.

### TUNEL assay

TUNEL staining was performed as described previously according to the manufacturers' protocols with minor modifications.^43^ Briefly, TUNEL assay was performed in 15-*μ*m-thick sections of paraffin-embedded brain tissue using *in situ* cell death detection kit (*In Situ* Cell Death Detection Kit, Fluorescein; Roche Applied Science, Penzberg, Upper Bavaria). The stained sections were photographed with an epifluorescence microscope (Olympus, Shinjuku-ku, Tokyo, Japan). The nuclei were stained with DAPI (blue), and the apoptotic cells appeared green. Two or three quadrants were selected from each section, and the number of DAPI-positive (blue) and TUNEL-positive (green) cells in each quadrant was counted. The percentage of TUNEL-positive cells was calculated using the formula green/blue × 100%.

### Stereotaxic AAV-CamKII*α*-SENP1-mCherry-3FLAG injection

For SENP1 expression in neurons, mouse SENP1 cDNA was inserted into AAV vector, pAAV-CaMKII*α*-MCS-mCherry-3FLAG (Obio Technology, Shanghai, China), under the control of CaMKIIα promoter and fused to mCherry-3FLAG. Vector construction and AAV production were performed by Obio Technology. The virus titer was 3.69 × 10^12^ genome copies/ml. For virus infection, 8-week-old male C57BL/6 mice were anesthetized and placed in a stereotaxic frame (RWD Life Science, San Diego, CA, USA). Stereotactic intracerebral injections of AAV into the primary somatosensory cortex, barrel field and secondary somatosensory cortex were performed, using the following coordinates (in mm from bregma) according to the mouse brain atlas: AP=+0.00 mm; L=+3.70 mm; DV=–2.50 mm. A total of 1.5 *μ*l of viral preparation was injected by microelectrodes connected with a microinjector pump (KDS 310; KD Scientific, Holliston, MA, USA) at a rate of 0.1 *μ*l/min. Microelectrodes were left in place for a further 10 min after each infusion to allow complete diffusion of AAV from the tip. Mice were allowed to recover for 4 weeks before conducting stroke model and the injection sites were examined at the end of the experiments. Brain slices from animals injected with the viruses were examined directly using fluorescence microscopy.

### *In vivo* sumoylation assay

After killing, CP tissue, as indicated by the dashed contour lines in Figure 1b, was extracted from freshly dissected mouse brain of MCAO or sham (equivalent area as MCAO) group using the micropunch method as previously described.^42^ They were then added to the SDS lysis buffer (50 mM Tris-HCl, 2% SDS, 40 mM DTT, 5% glycerol, pH 6.8), homogenized using a glass blender, boiled for 10 min at 95 °C, diluted with NP40 lysis buffer (150 mM NaCl, 1% NP40, 50 mM Tris-HCl, pH 7.4–8.0) containing 20 mM NEM and protease inhibitors, sonicated (5 s) and centrifuged (13 000 r.p.m., 8 min) to remove cell debris. The lysates were incubated with an elution buffer for 5 min at 100 °C and collected by centrifugation for SDS-PAGE analysis. Western blots were analyzed with an anti-SUMO1 antibody.

### Statistical analysis

Image analyses were performed using ImageJ software and statistical analysis performed with GraphPad Prism 5.01 (GraphPad Software Inc., La Jolla, CA, USA) statistical packages. Data are expressed as means±S.E.M. with statistical significance assessed by Student's *t*-test for two-group comparison or one-way analysis of variance for more than two groups. *P*-values <0.05 were considered to have statistically significant difference.
